# Probiotics and dental caries

**DOI:** 10.3402/mehd.v23i0.18582

**Published:** 2012-06-18

**Authors:** Eva Söderling

**Affiliations:** Institute of Dentistry, University of Turku, Turku, Finland

Caries is one of the most common infectious diseases in the world. Dental caries can be defined as destruction of the tissues of the tooth by bacterial fermentation of dietary carbohydrates. In this respect, some bacteria are considered more caries-promoting than others, as an example *Streptococcus mutans*. A common feature for caries-promoting bacteria is that they are acidogenic and aciduric.

The most common bacteria used as probiotics, lactobacilli and bifidobacteria, are, in theory, caries-promoting. They are excellent acid producers, they tolerate low pH-values, and they are frequently found in caries lesions. Since probiotics can be consumed several times a day and they are even given to infants, it is essential to know that they are safe.

All studies so far indicate that probiotics have rather beneficial than adverse effects on the caries risk. The best-studied probiotics *Lactobacillus rhamnosus* GG (LGG), *Lactobacillus reuteri*, and *Bifidobacterium lactis* BB-12 (BB-12) colonize poorly the oral cavity of adults (1–3). A recent study shows that BB-12 administered to infants twice a day showed poor retention to the teeth and oral mucosa of the infants (4). Transient colonization reduces the possible risks for oral health.

Little is known about the effects of the above probiotics on the composition of the oral flora. Short-term consumption of LGG, *L. reuteri*, and BB-12 have in some studies reduced counts of *S. mutans* (5, 6). Also, the amount of dental plaque (biofilm on the teeth) has been reduced by some probiotics. Dental plaque is not only a caries risk factor but also associated with periodontal diseases. A recent study showed that consumption of LGG and *L. reuteri* did not result in an increase in the plaque acid production potential, an important virulence factor of plaque (7).

There are only a few studies in which occurrence of caries has been studied in connection with consumption of probiotics. Näse et al. (5) found that LGG milk reduced caries occurrence in 3–4-years-old children. Milk with *L. rhamnosus* LB21 and fluoride reduced caries occurrence in school children (8). Reversals of root caries lesions were studied with *L. rhamnosus* LB21 milk w/wo fluoride – in that study, not only the fluoride-containing milks but also the probiotic milk had beneficial effects on root caries (9). Recently, caries occurrence was studied in 4-years-old children who had received BB-12 twice a day during infancy. The administration of the probiotic did not increase the caries occurrence (Taipale et al., unpublished results).

Clearly, long-term clinical studies with the disease occurrence as the primary outcome measure are needed to establish beneficial versus adverse effects of probiotics on oral health. Optimally, ‘old’ probiotics with proven benefits for general health could be also be used to benefit dental health.
